# Vinylphosphonium and 2-aminovinylphosphonium salts – preparation and applications in organic synthesis

**DOI:** 10.3762/bjoc.13.269

**Published:** 2017-12-15

**Authors:** Anna Kuźnik, Roman Mazurkiewicz, Beata Fryczkowska

**Affiliations:** 1 Department of Organic and Bioorganic Chemistry and Biotechnology, Silesian University of Technology, B. Krzywoustego 4, 44-100 Gliwice, Poland; 2 Biotechnology Center of Silesian University of Technology, B. Krzywoustego 8, 44-100 Gliwice, Poland; 3 Institute of Textile Engineering and Polymer Materials, University of Bielsko-Biala, Willowa 2, 43-309 Bielsko-Biala, Poland

**Keywords:** 2-aminovinylphosphonium salts, nucleophilic addition, phosphorus ylides, vinylphosphonium salts, Wittig reaction

## Abstract

The main synthetic routes towards vinylphosphonium salts and their wide applications in organic synthesis are discussed in this review. Particular attention is paid to the use of these compounds as building blocks for the synthesis of carbo- and heterocyclic systems after their prior transformation into the corresponding phosphorus ylides, followed by the intramolecular Wittig reaction with various types of nucleophiles containing a carbonyl function in their structures.

## Introduction

**Scheme 1:**
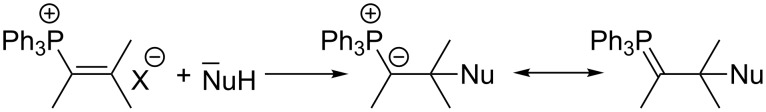
Generation of phosphorus ylides from vinylphosphonium salts.

Vinylphosphonium salts have been known for a long time, although significant interest in these compounds dates back to 1964, when Schweizer found that they can be converted to phosphorus ylides by the addition of a nucleophile to the carbon atom at the β-position of the vinyl group (Scheme 1) [1].

**Scheme 2:**
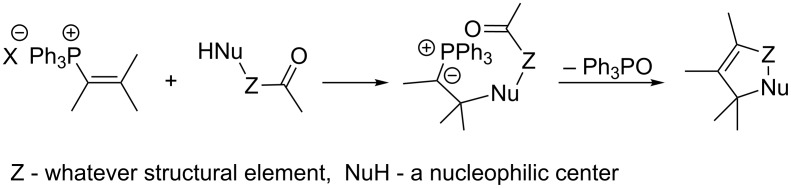
Intramolecular Wittig reaction with the use of vinylphosphonium salts.

Particularly widely used in organic synthesis are reactions involving the addition of a nucleophile containing a carbonyl group in its molecule to vinylphosphonium salt. A phosphorus ylide thus generated undergoes a subsequent intramolecular Wittig reaction, leading to a carbo- or heterocyclic ring closure (Scheme 2) [1–3]. This reaction can be considered as a general method for the synthesis of carbo- and heterocyclic systems. Schweizer's discovery became the basis for the wide use of vinylphosphonium salts in organic synthesis while stimulating the development of methods for the synthesis of those compounds.

Wide synthetic application, particularly for the synthesis of heterocycles found also 2-aminovinylphosphonium salts and their derivatives. The preparation and synthetic use of these compounds were presented by Drach, Brovarets and Smolii in a comprehensive review in 2002 [4]. In this paper we will discuss only the last reports on the synthesis and properties of 2-aminovinylphosphonium salts and their derivatives that were not included in the above-mentioned review article.

Increasing interest in phosphonium salts is also due to their use in drug design. It was demonstrated in the last decade that lipophilic cations having a triphenylphosphonium residue in the structure can be used as effective carriers of anticancer drugs, antioxidants, or functional probes into the mitochondria [5–8].

## Review

### 1. Synthesis of vinylphosphonium salts

#### 1.1. Alkylation of phosphines with alkyl halides

**Scheme 3:**
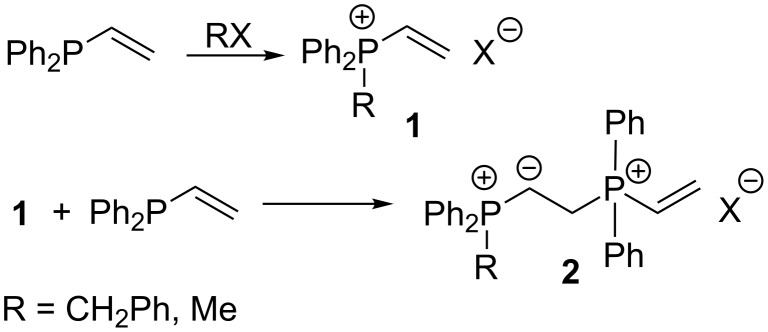
Alkylation of diphenylvinylphosphine with methyl or benzyl iodide.

One of the most common methods for the preparation of vinylphosphonium salts **1** is the quaternization of vinylphosphines with alkyl halides. Shutt and Trippett were able to alkylate vinylphosphines with methyl iodide or benzyl iodide, although attempts to use other alkyl halides, including benzyl bromide, ethyl bromoacetate and ethyl iodoacetate, in the presence of aprotic solvents provided only β-phosphonioylides **2** or polymeric phosphonium salts that were amorphous and unable to crystallize. Such products may be formed as a result of a subsequent Michael-like addition of the starting phosphine to the initially obtained vinylphosphonium salt **1** (Scheme 3). The formation of the expected salt was faster than the side addition reaction only in reactions with methyl iodide and benzyl iodide [9].

**Scheme 4:**
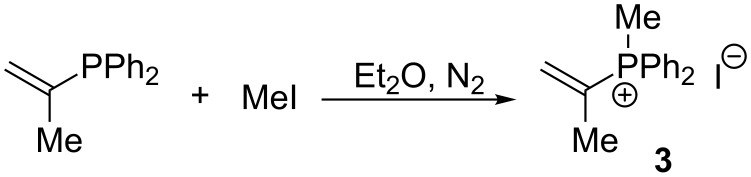
Methylation of isopropenyldiphenylphosphine with methyl iodide.

A similar alkylation of isopropenyldiphenylphosphine with methyl iodide in ether solution under a nitrogen atmosphere leading to isopropenylmethyldiphenylphosphonium iodide (**3**) in a yield of 97% was described by Schweizer and Wehman (Scheme 4) [10].

**Scheme 5:**
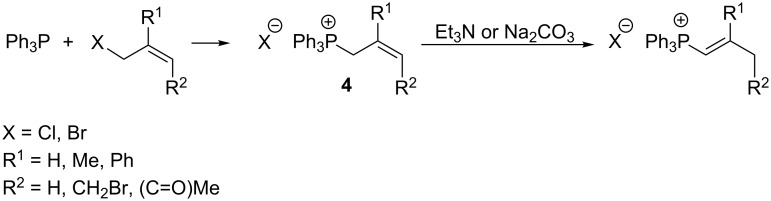
Alkylation of phosphines with allyl halide derivatives and subsequent isomerization of intermediate allylphosphonium salts.

Vinylphosphonium salts can also be prepared by alkylation of phosphines (usually triphenylphosphine) with allyl halide derivatives and isomerization of allylphosphonium salts **4** thus obtained under the influence of bases such as triethylamine or sodium carbonate (Scheme 5) [11–13].

**Scheme 6:**
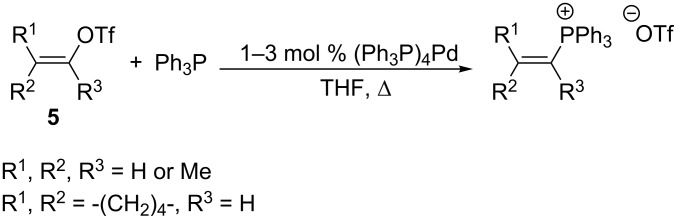
Alkylation of triphenylphosphine with vinyl triflates in the presence of (Ph_3_P)_4_Pd.

Vinyl halides are relatively less reactive alkylating agents. However, the use of readily available vinyl triflates **5** for the alkylation of triphenylphosphine in THF solution in the presence of a catalytic amount of (Ph_3_P)_4_Pd (1–3 mol %) gave the expected vinylphosphonium salts in a yield of 62–89% and a high stereoselectivity (Scheme 6) [14–15].

**Scheme 7:**
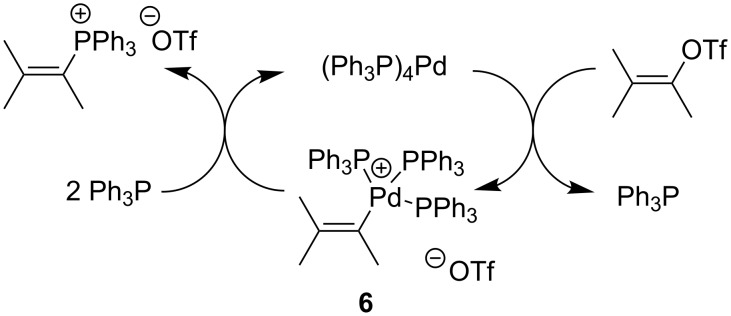
Mechanism of alkylation of triphenylphosphine with vinyl triflates in the presence of (Ph_3_P)_4_Pd as catalyst.

The proposed mechanism of this reaction is as described in Scheme 7 [14–15].

Oxidative addition of the vinyl triflate to the catalyst results in complex **6** that upon reductive elimination (an added phosphine) provides the vinylphosphonium salt and regenerates the Pd(0) catalyst (Scheme 7).

#### 1.2. β-Elimination of β-phenoxyalkyl- or α-haloalkylphosphonium salts

**Scheme 8:**
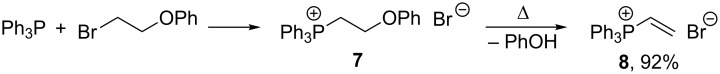
β-Elimination of phenol from β-phenoxyethyltriphenylphosphonium bromide.

Schweizer and Bach described the synthesis of vinyltriphenylphosphonium bromide (**8**) by heating a solution of the β-phenoxyethylphosphonium salt **7** in ethyl acetate. The final step of the reaction consisted in the β-elimination of the phenol molecule (Scheme 8) [16].

**Scheme 9:**
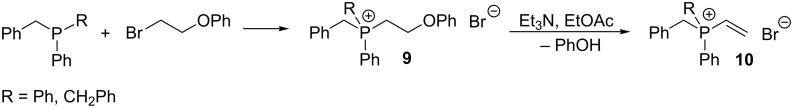
β-Elimination of phenol from β-phenoxyethylphosphonium salts in an alkaline environment.

A similar reaction using β-phenoxyethylphosphonium salts **9** derived from benzyldiphenylphosphine or dibenzylphenylphosphine required an alkaline environment and gave the expected vinylphosphonium salts **10** in good yields (Scheme 9) [16].

**Scheme 10:**
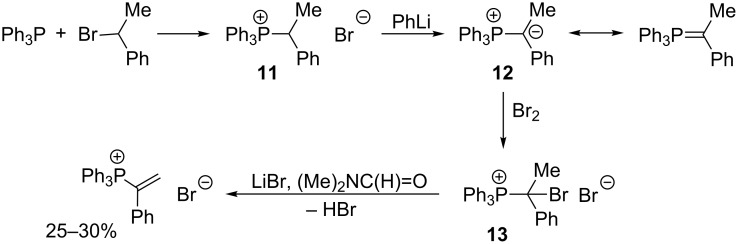
Synthesis and subsequent dehydrohalogenation of α-bromoethylphosphonium bromide.

Vinylphosphonium salt can also be synthesized by dehydrohalogenation of α-bromoethylphosphonium bromide **13** in the presence of lithium bromide in anhydrous dimethylformamide (Scheme 10). α-Bromoethylphosphonium salt **13** was obtained according to the three-step procedure, starting from the alkylation of triphenylphosphine with 1-bromoethylbenzene. The resulting phosphonium salt **11** was then deprotonated to the corresponding ylide **12**, which in the last step was subjected to bromination to give the expected α-bromoethylphosphonium bromide **13** [10].

#### 1.3. Peterson olefination of α-trimethylsilylphosphonium ylides with aldehydes

**Scheme 11:**
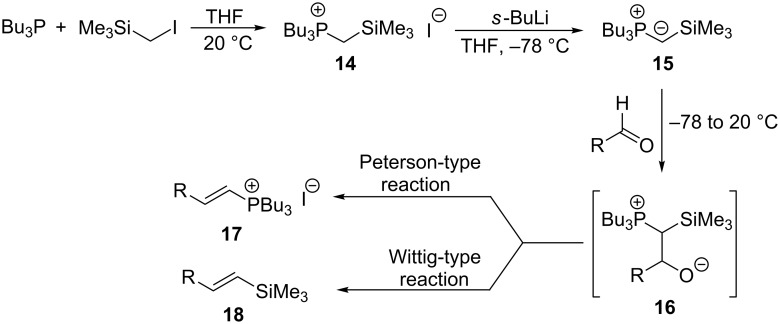
Synthesis of tributylvinylphosphonium iodides via Peterson-type olefination of α-trimethylsilylphosphonium ylides with aldehydes.

An interesting alternative pathway to vinylphosphonium salts, based on a Peterson-like olefination of α-trimethylsilyl phosphonium ylides **15**, was described by McNulty and Das and by Łukaszewicz et al. The commercially readily available iodomethyltrimethylsilane was reacted with tributylphosphine at room temperature to give the corresponding α-trimethylsilylphosphonium salt **14**. The latter salt was deprotonated in the presence of *s*-BuLi under kinetically controlled conditions, and the resulting ylide **15** reacted with aldehyde, providing tributylvinylphosphonium salt derivatives **17** in good yields via Peterson olefination through a silylated betaine **16**. The alternatively possible Wittig reaction to vinylsilane **18** can be considered as a side reaction (Scheme 11) [17–18].

The transformation shown in Scheme 11 was proven to be a general reaction with the possible participation of both electron-rich and electron-deficient aromatic aldehydes, giving the expected vinylphosphonium salts **17** with high yields and stereoselectivity, favoring the formation of an excess of *E-*isomers (Table 1) [17].

**Table 1:** Synthesis of vinylphosphonium salts by reaction of α-silyl ylides with aldehydes [17].

aldehyde	vinylphosphonium salt	*E*/*Z* ratio	yield [%]

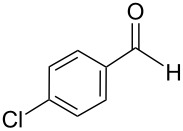	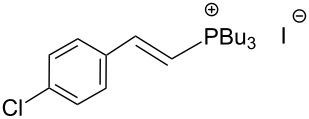	49:1	90
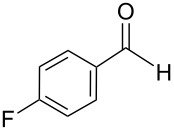	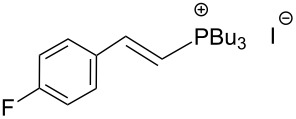	49:1	95
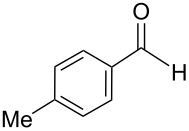	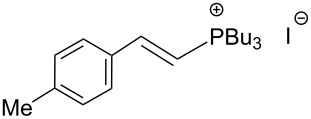	49:1	92
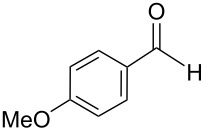	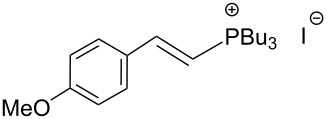	49:1	91
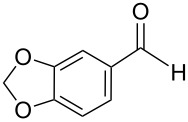	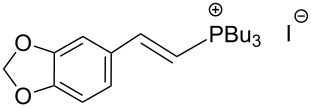	49:1	85
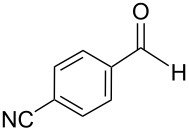	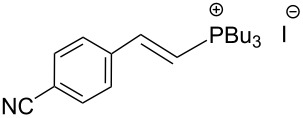	49:1	90
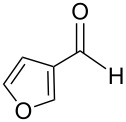	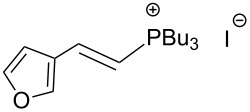	5:1	85
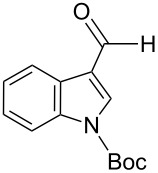	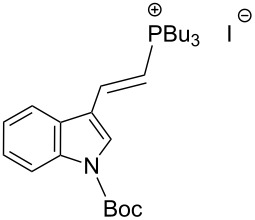	3:1	99
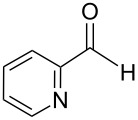	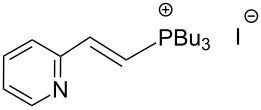	3:1	90
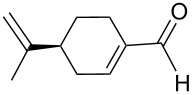	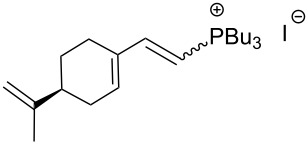	1:3	75
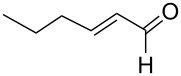	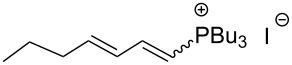	1.1:1	77

#### 1.4. Electrochemical oxidative addition of triphenylphosphine to cycloalkenes

**Scheme 12:**
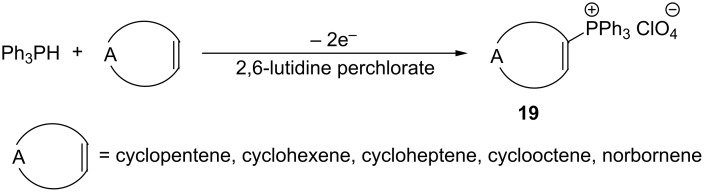
Synthesis of 1-cycloalkenetriphenylphosphonium salts by electrochemical oxidation of triphenylphosphine in the presence of cycloalkenes.

Another effective method for obtaining vinylphosphonium salts consists in the one-step electrochemical oxidation of triphenylphosphine in the presence of cycloalkenes. The synthesis of 1-cycloalkenetriphenylphosphonium salts **19** was carried out in the presence of 2,6-lutidine perchlorate and anhydrous potassium carbonate under a nitrogen atmosphere in dichloromethane solution on a graphite anode and a cathode of stainless steel at a constant current of 20 mA (Scheme 12). Depending on the cycloalkene used, the target vinylphosphonium salt was obtained in a yield of 53–66% [19].

**Scheme 13:**
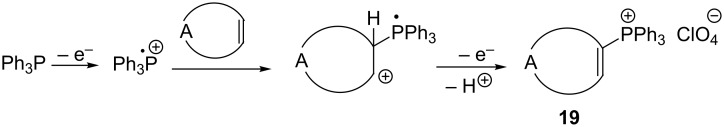
Suggested mechanism for the electrochemical synthesis of 1-cycloalkenetriphenylphosphonium salts.

Since the oxidation potential of cycloalkene is higher than that for triphenylphosphine, the suggested mechanism of formation of the final 1-cycloalkenetriphenylphosphonium salts **19** is analogous to the reactions of the radical cation of triphenylphosphine with other nucleophiles (Scheme 13) [19].

#### 1.5. Triphenylphosphine addition to a triple carbon–carbon bond of acetylenedicarboxylic acid esters

**Scheme 14:**
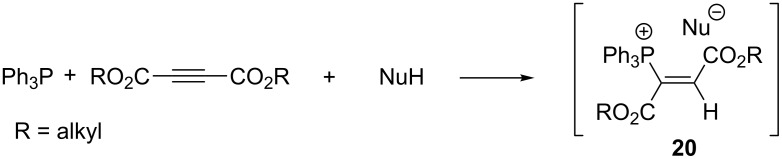
Generation of α,β-(dialkoxycarbonyl)vinylphosphonium salts by addition of triphenylphosphine to acetylenedicarboxylates in the presence of nucleophiles.

The addition of triphenylphosphine to acetylenedicarboxylic acid esters in the presence of a nucleophile is a frequently used method for the generation of unstable, highly reactive α,β-(dialkoxycarbonyl)vinylphosphonium salts **20** (Scheme 14) that are commonly used in further reactions without prior isolation [20–28]. Their generation and subsequent transformations are comprehensively discussed in section 2.3.2.

#### 1.6. Synthesis and structure of 2-aminovinylphosphonium salts

**Scheme 15:**
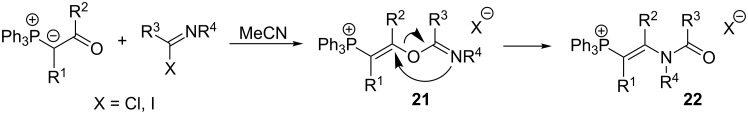
Synthesis of 2-(*N*-acylamino)vinylphosphonium halides by imidoylation of β-carbonyl ylides with imidoyl halides.

**1.6.1. 2-(*N*-Acylamino)vinylphosphonium salts by imidoylation of β-carbonyl ylides:** 2-(*N*-Acylamino)vinylphosphonium halides **22** can be prepared by imidoylation of β-carbonyl phosphorus ylides with imidoyl halides in acetonitrile (Scheme 15). The obtained salts **22** are stable, crystalline compounds that are usually synthesized in good yields according to one of the three procedures described by Mazurkiewicz et al. (Table 2) [29–31]. The first method (procedure A) consists in the addition of the ylide to the solution of the imidoyl chloride (Scheme 15, X = Cl). The expected 2-(*N*-acylamino)vinylphosphonium salts **22** are formed in acetonitrile by a rearrangement of the primary *O*-imidoylation reaction product **21** (Scheme 15).

**Table 2:** Synthesis of 2-(*N*-acylamino)vinylphosphonium salts [31].

ylide		imidoylating agent			2-(*N*-acylamino)vinylphoshonium salts		

R^1^	R^2^	R^3^	R^4^	X	procedure	yield [%]	mp [°C]

H	H	Me	Ph	I	B	91	133–134
H	H	Ph	Me	Cl	A	71	238–239
H	H	Ph	Me	Br	C	71	242–243
H	H	Ph	PhCH_2_	Cl	A	64	273–275
H	Me	Ph	Me	Cl	A	66	140–141
H	Me	Ph	Me	I	B	85	196–198
H	Me	Ph	Me	Br	C	80^a^	196–198
H	Me	Ph	Ph	Cl	A	72	192–193
H	Me	Ph	PhCH_2_	Cl	A	87	175–177
H	Me	(CH_2_)_5_		Br	C	79	205.5–206
Me	H	Ph	Me	Cl	A	99	resin
Me	H	(CH_2_)_4_		Br	C	62	resin

^a^A mixture of stereoisomers in the ratio of 68:32.

The second method (procedure B) is based on the reaction of ylides with imidoyl iodides that are synthesized in situ from the corresponding imidoyl chlorides via the exchange of chlorine for iodine in the presence of sodium iodide (Scheme 15, X = I).

**Scheme 16:**
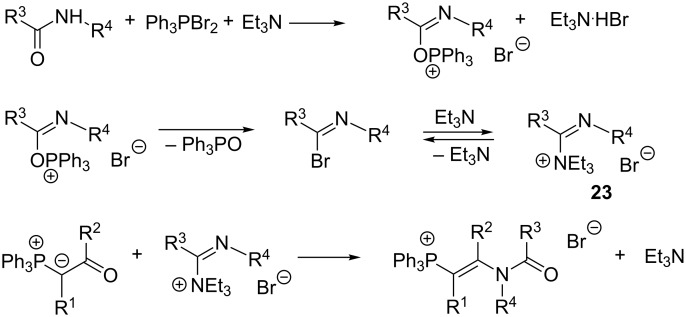
Imidoylation of β-carbonyl ylides with imidoyl halides generated in situ.

In the case of unstable or inaccessible imidoyl halides (Scheme 16) it is also possible to use an imidoylating agent in this reaction that is generated in situ from the amide or lactam by using dibromotriphenylphosphorane in the presence of triethylamine (procedure C). Spectral evidences were provided that both imidoyl bromide and *N*,*N*,*N*,*N*’-tetrasubstituted amidinium salt **23** could act in this reaction as an effective imidoylating agent [31].

**Scheme 17:**
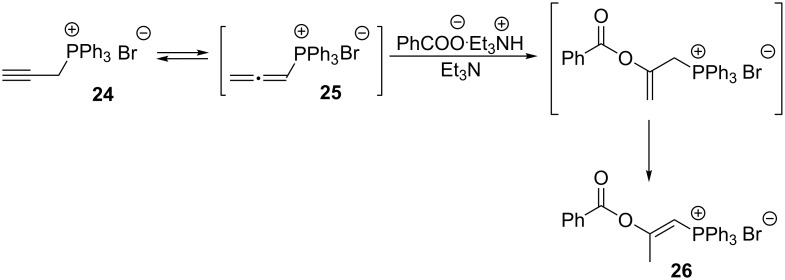
Synthesis of 2-benzoyloxyvinylphosphonium bromide from 2-propynyltriphenylphosphonium bromide.

**1.6.2. Vinylphosphonium and 2-aminovinylphosphonium salts via the addition of nucleophiles to 2-propynylphosphonium salts:** In 1969 Stirling and Appleyard only postulated that the reaction between the benzoate anion and 2-propynyltriphenylphosphonium bromide (**24**) led to a 2-benzoyloxyvinylphosphonium salt **26** (Scheme 17) [32].

**Scheme 18:**
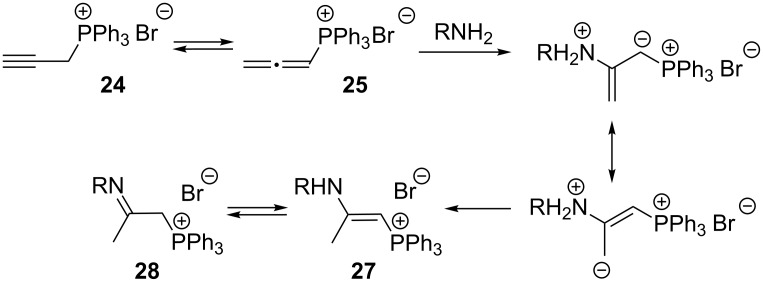
Synthesis of 2-aminovinylphosphonium salts via nucleophilic addition of amines to 2-propynyltriphenylphosphonium bromide.

Several years later, Schweizer et al. discovered that the nucleophilic addition of amines to 2-propynyltriphenylphosphonium bromide (**24**) involved propadienylphosphonium bromide (**25**) – the tautomeric form of the starting phosphonium salt. The same authors carried out a series of nucleophilic additions of amines and hydrazine derivatives to **24** to obtain products with the proposed structure of 2-aminovinylphosphonium salts **27** in equilibrium with their tautomeric imine form **28** (Scheme 18). Depending on the nature of the substituent R, the enamine or the imine form predominated (Table 3) [33].

**Table 3:** Products of reaction of 2-propynyltriphenylphosphonium bromide with amines [33].

product	R	**27**:**28** molar ratio	yield [%]	product	R	**27**:**28** molar ratio	yield [%]

**28a**	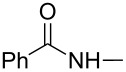	0:100	86	**27h**	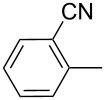	>99:trace	60.5
**28b**	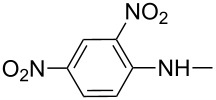	0:100	81	**27i**	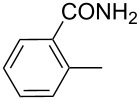	>99:trace	40
**28c**	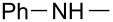	0:100	60.5	**27j**	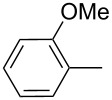	100:1	94
**27d**	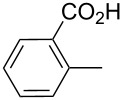	6:1	70	**27k**	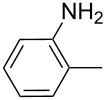	100:1	59
**27e**	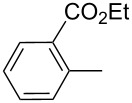	5:1	74	**27l**	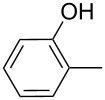	100:1	92
**27f**	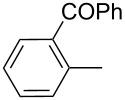	3:1	95	**27m**	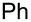	100:1	88
**27g**	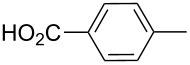	>99:trace	78	**27n**	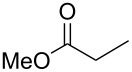	100:1	31

**Scheme 19:**
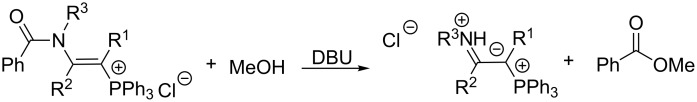
Deacylation of 2-(*N*-acylamino)vinylphosphonium chlorides to 2-aminovinylphosphonium salts.

In 2004 Mazurkiewicz and Fryczkowska discovered that the same type of compounds can be obtained in good or even very good yields by deacylation of 2-(*N*-acylamino)vinylphosphonium salts with methanol in the presence of DBU (Scheme 19) [34–35].

**Scheme 20:**
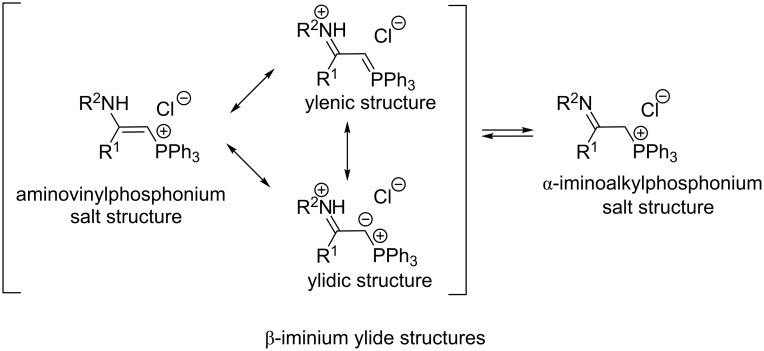
Resonance structures of 2-aminovinylphosphonium salts and tautomeric equilibrium between aminovinylphosphonium and α-iminoalkylphosphonium cations.

Spectroscopic properties (IR, ^1^H and ^13^C NMR) and X-ray data of the obtained 2-aminovinylphosphonium salts corresponded to the enamine tautomeric form with the domination of β-iminium ylide resonance structures. Furthermore, the authors did not observe an imine tautomeric form of the synthesized compounds as described by Schweizer (^1^H NMR). The isotopic exchange of acidic protons using a D_2_O solution in CD_3_CN revealed that the isotopic exchange of a proton in the α-position was possible only in the presence of a strong base, such as, for example, DBU [34–36]. In the case of a real tautomeric equilibrium between aminovinylphosphonium and α-iminoalkylphosphonium cations, the isotopic exchange should occur easily (Scheme 20).

**Scheme 21:**
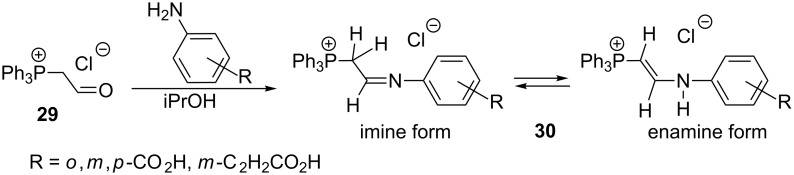
Synthesis of 2-aminovinylphosphonium salts by reaction of (formylmethyl)triphenylphosphonium chloride with amines.

Borodkin et al. have recently reported a new method for the synthesis of 2-aminovinylphosphonium salts **30** by reaction of (formylmethyl)triphenylphosphonium chloride (**29**) with aromatic amines in isopropanol in yields of 47–91% (Scheme 21). The initially obtained imine form of the product underwent tautomerization to a more stable enamine form, usually in *E*-configuration. The obtained compounds, particularly the derivative containing a carboxylic group in *ortho* position of the aromatic ring, exhibited antimicrobial activity [37].

### 2. Vinylphosphonium salts in organic synthesis

#### 2.1. Vinylphosphonium salts in the intermolecular Wittig reaction

**Scheme 22:**
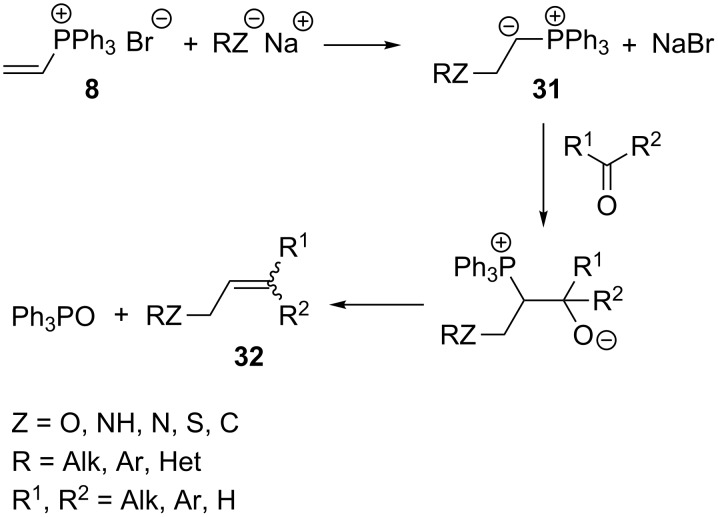
Generation of ylides by reaction of vinyltriphenylphosphonium bromide with nucleophiles and their subsequent intermolecular Wittig reaction with aldehydes or ketones.

Schweizer et al. developed a general method for the generation of phosphorus ylides **31** by reaction of a variety of nucleophiles with vinyltriphenylphosphonium bromide (**8**). The ylides were then subjected to an intermolecular Wittig reaction with aldehydes or ketones without isolation. The anionic forms of the nucleophilic agents used in these reactions were usually prepared by reaction of sodium hydride or sodium ethoxide with a proper nucleophile, such as diethylamine, piperidine, pyrrole, ethanol, *p*-toluenesulfonamide, thiophenol and others. Depending on the reactants used, *Z-* or *E-*stereoisomers of the products **32** were obtained, but most commonly the reactions resulted in a mixture of stereoisomers (Scheme 22). The yield of the reaction was dependent on the kind of substrate and ranged from 14 to 68% [38].

**Scheme 23:**
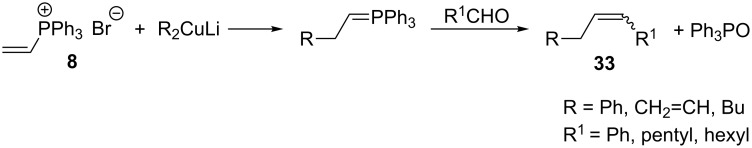
Intermolecular Wittig reaction with the use of vinylphosphonium bromide and organocopper compounds as carbon nucleophiles.

The reaction involving organocopper compounds as carbon nucleophiles (R_2_CuLi, where R = vinyl, butyl, phenyl) is another way of using vinylphosphonium bromide **8** in the intermolecular Wittig reactions (Scheme 23). Depending on the kind of substituent R and the aldehyde used, the yield of obtained compounds **33** was in the range of 25–80%. The reaction predominantly provided the *Z*-isomer of the alkene [39].

**Scheme 24:**
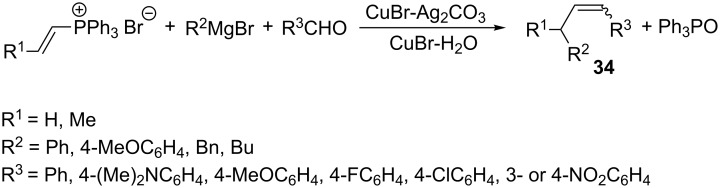
Intermolecular Wittig reaction with the use of ylides generated from vinylphosphonium bromides and Grignard reagents.

An interesting pathway of generating ylides from vinylphosphonium salts turned out to be the reaction of the latter compounds with Grignard reagents in the presence of CuBr·H_2_O or CuBr·Ag_2_CO_3_ (Scheme 24). The subsequent Wittig reaction allowed to obtain substituted alkenes **34** in a yield of 68–94% and in a good stereoselectivity. The configuration of products depended on the nature of the substituent in the phenyl group of the aldehyde. Electron-donating substituents favored the formation of *E*-isomers, while the presence of electron-withdrawing substituents made the formation of *Z*-isomers more favorable [40].

**Scheme 25:**
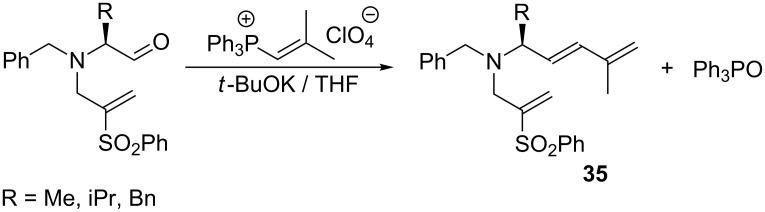
Direct transformation of vinylphosphonium salts into ylides in the presence of potassium *tert*-butoxide and their subsequent Wittig reaction with aldehydes.

Vinylphosphonium salts can also be directly converted into the corresponding ylides by potassium *tert*-butoxide and subjected to the Wittig reaction as described by Yamamoto et al. (Scheme 25). The transformation resulted in polyenes **35** of *Z-*configuration in a relatively low yield of 10–36% [41].

#### 2.2. Vinylphosphonium salts in the intramolecular Wittig reaction

**Scheme 26:**
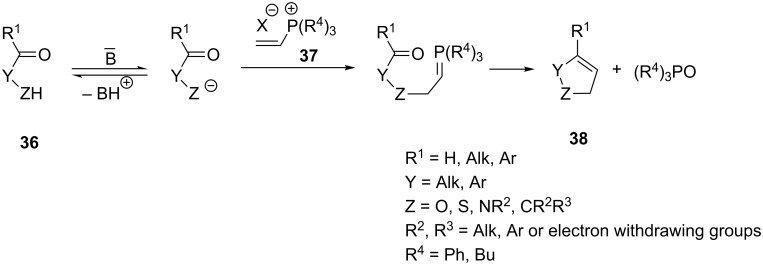
A general method for synthesis of carbo- and heterocyclic systems by the intramolecular Wittig reaction from vinylphosphonium halides and nucleophiles containing carbonyl function in the molecule.

As was already mentioned, in 1964 Schweizer provided a general method for preparing carbo- and heterocyclic compounds **38** using vinylphosphonium salts [1–2]. The method consisted in the reaction of oxygen, nitrogen and carbon nucleophiles **36** containing a carbonyl group in the molecule with vinylphosphonium halides **37** (Scheme 26). In the following chapter this general method for preparing a variety of carbo- and heterocyclic systems using various types of nucleophilic agents is discussed in detail.

**Scheme 27:**
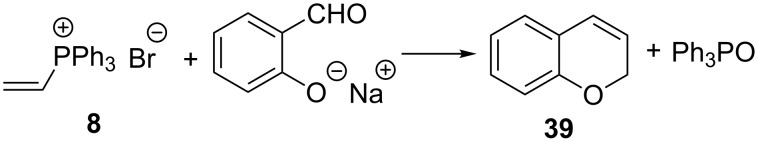
Synthesis of 2*H*-chromene by reaction of vinyltriphenylphosphonium bromide with sodium 2-formylphenolate.

**2.2.1. Reactions with oxygen nucleophiles:** In 1964 Schweizer reported the synthesis of 2*H*-chromene (**39**) by reaction of vinyltriphenylphosphonium bromide (**8**) with salicylaldehyde sodium salt in a yield of 62–71%, depending on the reaction conditions (Scheme 27) [1].

**Scheme 28:**
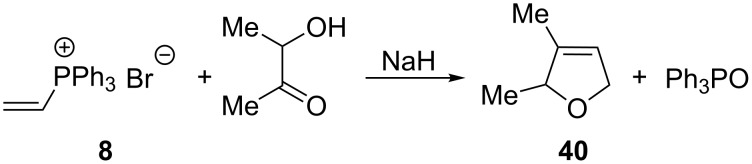
Synthesis of 2,5-dihydro-2,3-dimethylfuran by reaction of vinylphosphonium bromide with 3-hydroxy-2-butanone in the presence of sodium hydride.

In a similar reaction with the use of 3-hydroxy-2-butanone, 2,5-dihydro-2,3-dimethylfuran (**40**) was obtained in a yield of 89% (Scheme 28) [1].

**Scheme 29:**
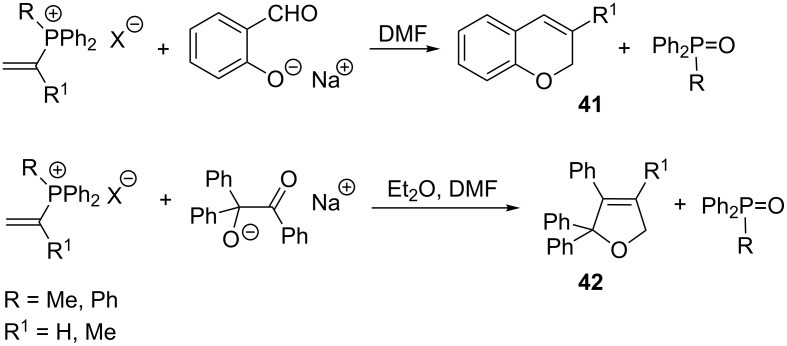
Synthesis of 2*H*-chromene and 2,5-dihydrofuran derivatives in the intramolecular Wittig reaction with the use of vinylphosphonium salts and an appropriate oxygen nucleophile containing a carbonyl group.

Several years later, Schweizer et al. applied analogous reaction conditions to the synthesis of 3-substituted derivative of 2*H*-chromene **41** and 2,5-dihydrofuran derivative **42** in yields in the range of 30–58% and 36–71%, respectively (Scheme 29) [42].

**Scheme 30:**
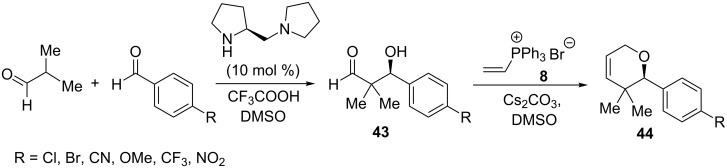
Enantioselective synthesis of 3,6-dihydropyran derivatives from vinylphosphonium bromide and enantiomerically pure oxygen nucleophile.

An interesting example of the application of ylides derived from vinylphosphonium salts in the enantioselective synthesis of pyran derivatives was reported by Ley et al. in 2010. β-Hydroxyaldehyde **43** as the oxygen nucleophile was obtained here in the asymmetric aldol condensation of 2-methylpropanal with an aromatic aldehyde using a chiral amine as the catalyst. The attack of the hydroxy group of the resulting enantiomerically pure oxygen nucleophile **43** on vinyltriphenylphosphonium bromide (**8**) in the presence of a base, providing an ylide as an intermediate, followed by the intramolecular Wittig reaction gave the corresponding 3,6-dihydropyran derivatives **44** in yields of 34–56% and a high enantioselectivity of 95–98% (Scheme 30) [43]. Pyran derivatives are structural elements of many natural biologically active compounds [44–47].

**Scheme 31:**
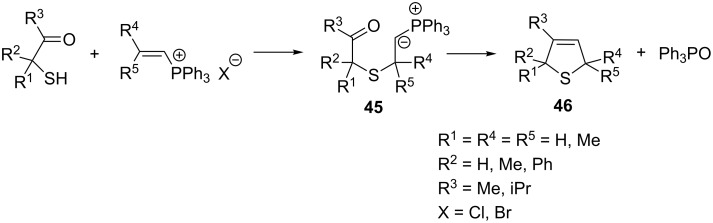
Synthesis of 2,5-dihydrothiophene derivatives in the intramolecular Wittig reaction from vinylphosphonium salts and sulfur nucleophiles.

**2.2.2. Reactions with sulfur nucleophiles:** The generation of phosphorus ylides in the reaction of vinylphosphonium salts with sulfur nucleophiles was reported by McIntosh et al. (Scheme 31). Phosphorus ylides **45** thus obtained were then converted to alkylated derivatives of 2,5-dihydrothiophene **46** as a result of the intramolecular Wittig reaction. The yields of the product ranged between 6 and 95% [13].

**Scheme 32:**
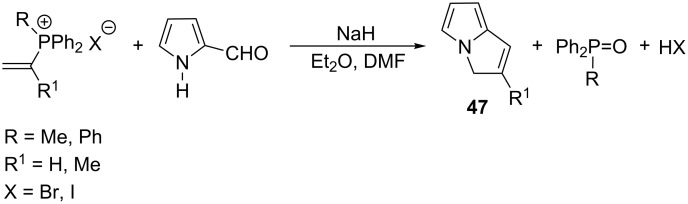
Synthesis of bicyclic pyrrole derivatives in the reaction of vinylphosphonium halides and 2-pyrrolocarbaldehyde.

**2.2.3. Reactions with nitrogen nucleophiles:** The reaction of vinylphosphonium salt with 2-pyrrolocarbaldehyde in the presence of sodium hydride as described by Schweizer et al. provided bicyclic pyrrole derivatives **47** in 25–87% yields (Scheme 32) [2,42].

**Scheme 33:**
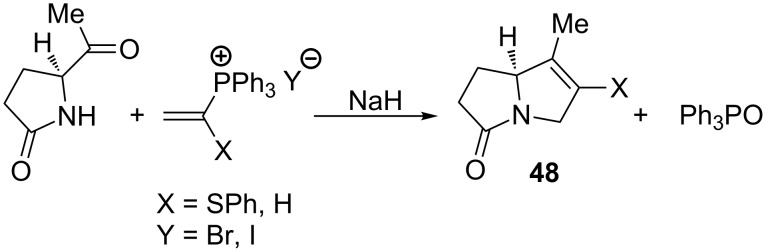
Stereoselective synthesis of bicyclic 2-pyrrolidinone derivatives in the reaction of vinylphosphonium halides and 5-acetyl-2-pyrrolidinone.

Similarly, Hewson and co-workers synthesized bicyclic 2-pyrrolidinone derivatives **48** from 5-acetyl-2-pyrrolidinone in a yield of 40–68% (Scheme 33) [48].

**Scheme 34:**
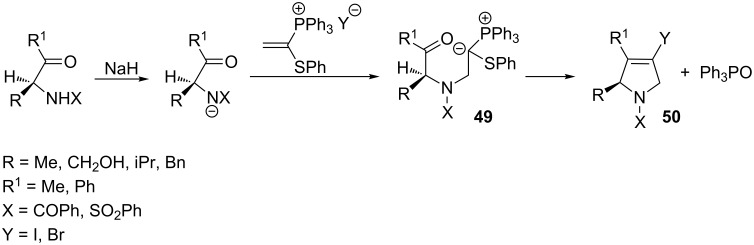
Stereoselective synthesis of 3-pyrroline derivatives in the intramolecular Wittig reaction from vinylphosphonium salts and nitrogen nucleophiles.

In 1994 Burley and Hewson reported a reaction of vinylphosphonium salt with nitrogen nucleophiles, obtained by deprotonation of β-ketosulfonamides or β-ketoamides with sodium hydride. The generated ylides **49** were then cyclized in the intramolecular Wittig reaction to give 3-pyrroline derivatives **50** in yields of 55–90%. It is worth emphasizing that enantiomerically pure 3-pyrroline derivatives were produced from enantiomerically pure β-ketoamides (Scheme 34) [49].

**Scheme 35:**
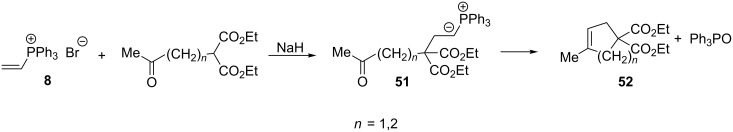
Synthesis of cyclic alkenes in the intramolecular Wittig reaction from vinylphosphonium bromide and ketoesters as precursors of carbon nucleophiles.

**2.2.4. Reactions with carbon nucleophiles:** The first reaction of vinylphosphonium salts with carbon nucleophiles was reported by Schweizer and O'Neill in 1965. The authors used vinyltriphenylphosphonium bromide (**8**) and ketoesters in the presence of sodium hydride to give the expected ylides **51**. A subsequent intramolecular attack of the ylidic carbon atom on the carbonyl group in the Wittig reaction provided 5- or 6-membered alkenes **52** in yields of 51–69% (Scheme 35) [3].

**Scheme 36:**
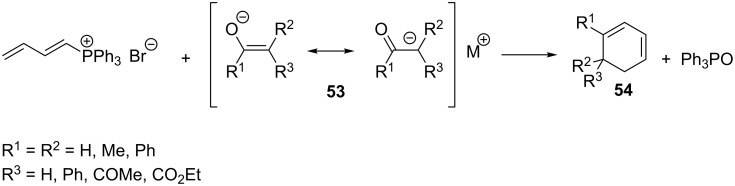
Synthesis of 1,3-cyclohexadienes by reaction of 1,3-butadienyltriphenylphosphonium bromide with enolate anions.

Another example of the use of the enolate anion as a carbon nucleophilic agent was reported by Fuchs, who obtained 1,3-cyclohexadienes **54** by reaction of 1,3-butadienyltriphenylphosphonium bromide with enolate anions **53** derived from aldehydes or ketones in yields of 35–57% (Scheme 36). The nucleophilic attack here was directed at position 4 of the diene [11].

**Scheme 37:**
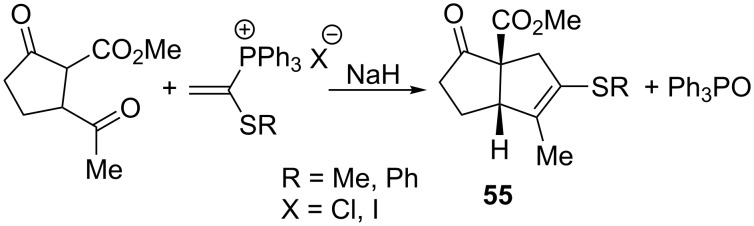
Synthesis of bicyclo[3.3.0]octenes by reaction of vinylphosphonium salts with cyclic diketoester.

Hewson and MacPherson performed a reaction of vinylphosphonium salts with a cyclic diketoester in the presence of sodium hydride which resulted in bicyclo[3.3.0]octenes **55** in 82–97% yield (Scheme 37) . The latter compounds were further used in the synthesis of natural compounds [50].

#### 2.3. Other synthetic uses of vinylphosphonium salts

**2.3.1. Deprotonated 2-aminovinylphosphonium salts in the Wittig reaction:** 2-Aminovinylphosphonium salts, which are the addition products of amines to 2-propynylphosphonium bromide [33] or can be obtained by deacylation of 2-(*N*-acylamino)vinylphosphonium salts [34–35], have been found to have several interesting synthetic applications, although they have only relatively recently become known.

**Scheme 38:**
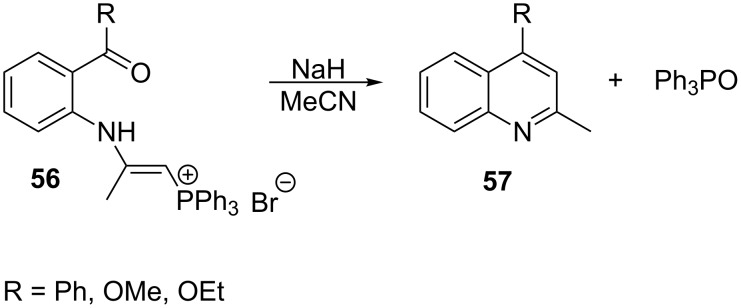
Synthesis of quinoline derivatives in the intramolecular Wittig reaction from 2-(2-acylphenylamino)vinylphosphonium salts.

Deprotonated 2-aminovinylphosphonium salts can be employed in Wittig reactions with carbonyl compounds. Thus the intramolecular Wittig reaction of 2-(2-acylphenylamino)vinylphosphonium salts **56** in the presence of sodium hydride provided quinoline derivatives **57** in 41–64% yield (Scheme 38) [33].

**Scheme 39:**
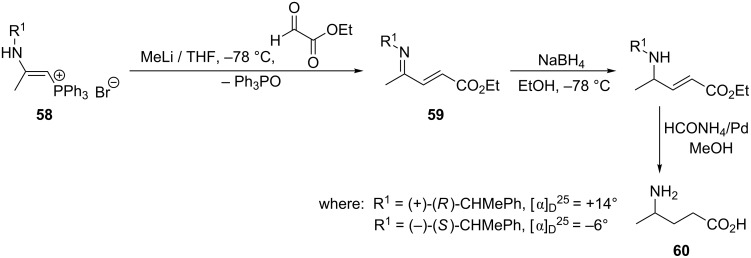
Stereoselective synthesis of γ-aminobutyric acid in the intermolecular Wittig reaction from chiral 2-aminovinylphosphonium bromides and glyoxylic acid esters.

Palacios et al. used 2-aminovinylphosphonium salts **58** for the synthesis of biologically active compounds or their precursors, e.g., azadiene **59**, which is a precursor of the important γ-aminobutyric acid (**60**) in the stereoselective synthesis thereof. The intermolecular Wittig reaction of these salts **58** with glyoxylic acid esters gave a dextro or levo γ-aminobutyric acid with values of specific rotation +14° and −6°, respectively, depending on the kind of chiral substituent R^1^ that was used (Scheme 39) [51].

γ-Aminobutyric acid is a major neurotransmitter used to treat epilepsy [52]. Analogs of this acid displaying antitumor activity, such as hapalosin, dolastatins, and caliculins were found in natural marine products [53–55].

**Scheme 40:**
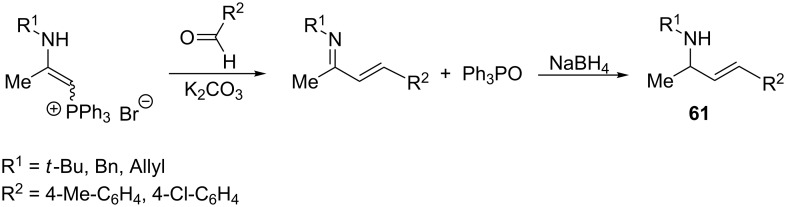
Synthesis of allylamines in the intermolecular Wittig reaction from 2-aminovinylphosphonium bromides with aldehydes or ketones.

Palacios et al. also described the Wittig reactions of 2-aminovinylphosphonium salts with aldehydes and ketones in THF in the presence of K_2_CO_3_ leading to allylamines **61**, which are an important class of compounds due to their biological activities (Scheme 40) [56]. They are used, inter alia, as chemotherapeutic agents, enzyme inhibitors, and antifungal compounds [57–59]. The allylamine structure exists in numerous natural products [60]. Moreover, allylamines are widely used in the synthesis of compounds such as β-aminohydroxylamines, β- and γ-amino acids, pseudopeptides, spermidine derivatives or five- and six-membered heterocyclic systems [56].

**Scheme 41:**
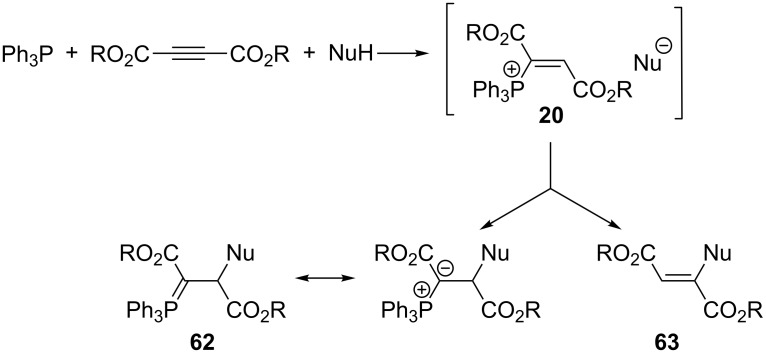
A general route towards α,β-di(alkoxycarbonyl)vinylphosphonium salts and their subsequent possible transformations.

**2.3.2. In situ generation of α,β-di(alkoxycarbonyl)vinylphosphonium salts and their further transformations:** A significant number of reactions of acetylenedicarboxylic acid esters with triphenylphosphine and nucleophiles of a general NuH structure leading to reactive α,β-di(alkoxycarbonyl)vinylphosphonium salts **20** have been described (Scheme 41). Depending on the structure of the nucleophile used, the salts either convert into resonance-stabilized, relatively stable ylides **62** or undergo intramolecular nucleophilic substitution with the triphenylphosphine departure to form products **63**. The latter usually undergo further cyclization involving one of the alkoxycarbonyl groups. Ylides **62** also undergo some further transformations in certain cases.

**Formation of resonance-stabilized phosphorus ylides and their further transformations:** Phosphorus ylides are organic compounds that are being used increasingly more often in the synthesis of many naturally occurring compounds which exhibit biological and pharmaceutical activity [61].

**Scheme 42:**
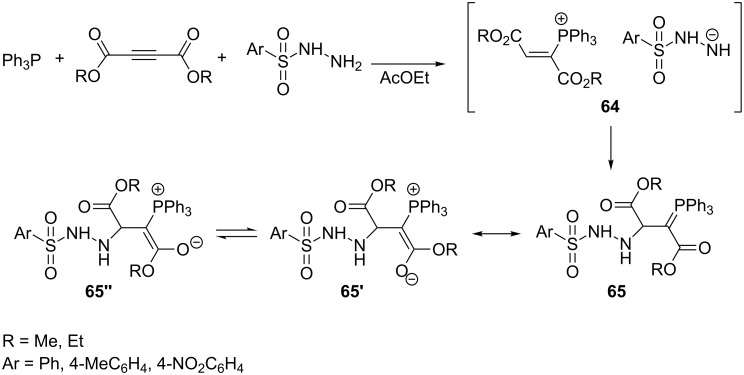
Generation of resonance-stabilized phosphorus ylides via the reaction of triphenylphosphine with dialkyl acetylenedicarboxylates and arylsulfonic hydrazides.

A typical example of the generation of resonance-stabilized phosphorus ylides **65** comprises triphenylphosphine, dialkyl acetylenedicarboxylate, and arylsulfonic hydrazides providing the corresponding ylides in high yields of 90 to 95% as two rotational isomers **65’** and **65’’** (Scheme 42). The intermediate product, formed by the addition of triphenylphosphine to dialkyl acetylenedicarboxylate, is protonated here by arylsulfonic hydrazide as an NH-acid. The deprotonated form of the NH-acid as a nitrogen nucleophile then attacks the β-position of the vinylphosphonium salt **64**, resulting in the expected resonance-stabilized ylide **65** [62].

**Scheme 43:**
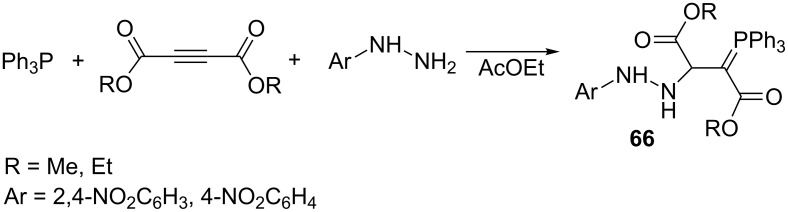
Synthesis of resonance-stabilized phosphorus ylides in the reaction of triphenylphosphine, dialkyl acetylenedicarboxylates, and aryl hydrazines.

The corresponding ylides **66** were also obtained in high yields of 90–94% in an analogous reaction by the use of aryl hydrazines (Scheme 43) [62].

**Scheme 44:**
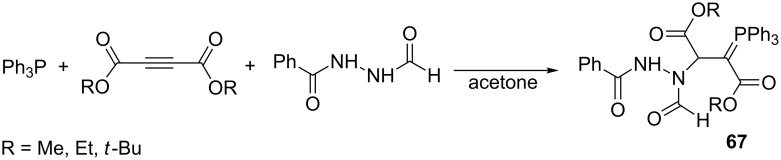
Synthesis of resonance-stabilized phosphorus ylides via the reaction of triphenylphosphine with dialkyl acetylenedicarboxylates and *N*’-formylbenzohydrazide.

The same type of reaction but with the use of *N*’-formylbenzohydrazide as an NH-acid leading to stabilized phosphorus ylides **67** with high yields was reported in 2012 by Hassanabadi et al. (Scheme 44) [26].

**Scheme 45:**
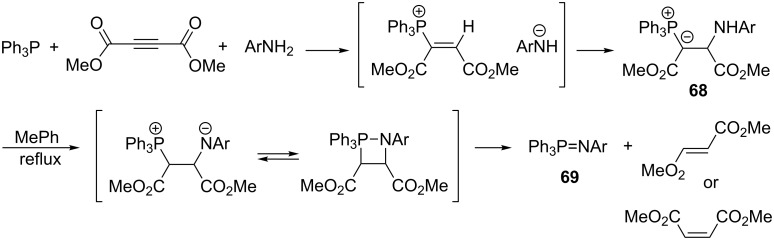
Generation of resonance-stabilized phosphorus ylides in the reaction of acetylenedicarboxylate, triphenylphosphine, and amines and their subsequent transformation into aryliminophosphoranes.

In an analogous manner, Yavari and co-workers obtained resonance-stabilized phosphorus ylides **68** via the reaction of acetylenedicarboxylic acid diester with triphenylphosphine and aromatic amines such as aniline, *p*-toluidine, 4-acetylaniline, 4-bromoaniline, 4-nitroaniline, 1-naphthylamine, 2-aminopyridine, and 2-amino-5-bromopyridine in dichloromethane at room temperature in yields of 96–98% (Scheme 45). The resulting ylides **68** were converted into aryliminotriphenylphosphoranes **69** (97–98%) and dimethyl fumarate or maleate at the boiling temperature of toluene [63].

**Scheme 46:**
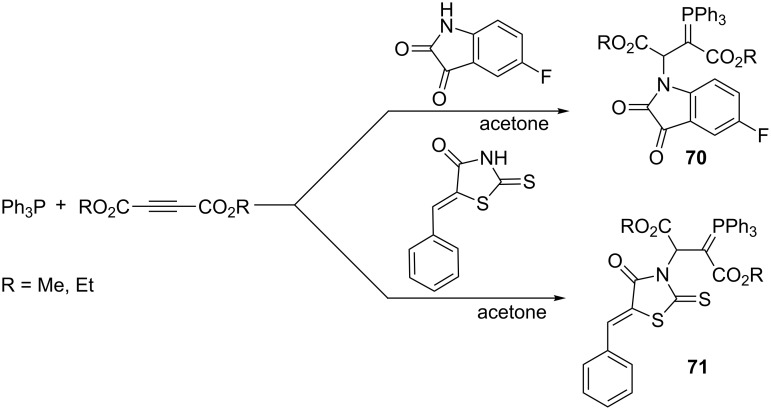
Synthesis of resonance-stabilized phosphorus ylides via the reaction of dialkyl acetylenedicarboxylates with triphenylphosphine and 2,3-indoledione or 4-thiazolidine-2-thione derivatives.

Recently, similar syntheses of resonance-stabilized ylides were described using 5-fluoro-2,3-indoledione or 4-thiazolidine-2-thione derivative as NH-acids and precursors of nitrogen nucleophiles. The expected resonance-stabilized phosphorus ylides **70** and **71** were synthesized in yields of 92–95% (Scheme 46) [28].

**Scheme 47:**
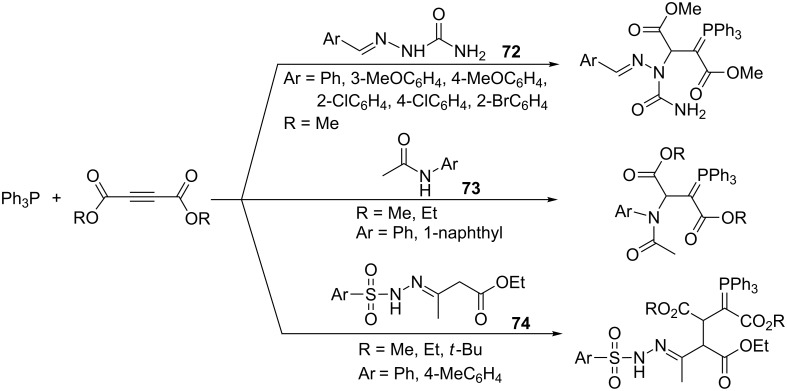
Synthesis of resonance-stabilized ylides derived from semicarbazones, aromatic amides, and 3-(arylsulfonylhydrazono)butanoates, respectively.

Between 2007 and 2013 Anary-Abbasinejad et al. reported applications in similar syntheses of semicarbazones **72** [64] or aromatic amides **73** [24] as the NH-acids and of 3-(arylsulfonylhydrazono)butanoates **74** as the CH-acids [27]. Diastereoselectivity of the reaction was found in the latter case (Scheme 47) [27].

**Scheme 48:**
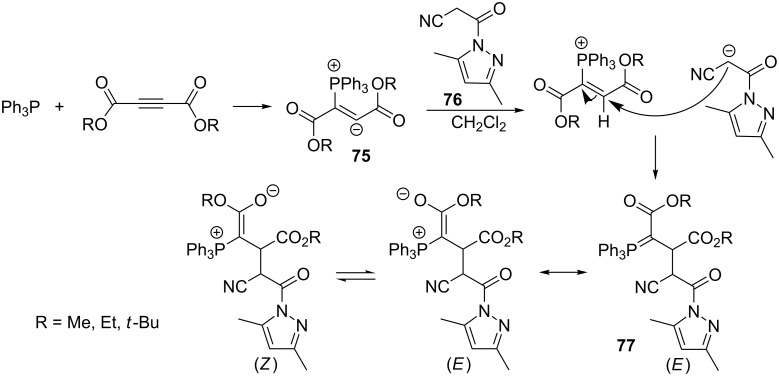
Synthesis of resonance-stabilized ylides via the reaction of triphenylphosphine with dialkyl acetylenedicarboxylates and 3-oxopropanenitrile derivative.

The generation of two isomers of phosphorus ylides was carried out via the addition of triphenylphosphine to DAAD (dialkyl acetylenedicarboxylate), followed by protonation of the intermediate adduct **75** by 3-(3,5-dimethylpyrazol-1-yl)-3-oxopropanenitrile (**76**) as a CH-acid. The expected resonance-stabilized ylides **77** were obtained in high yields of 90–95% (Scheme 48) [65].

**Scheme 49:**
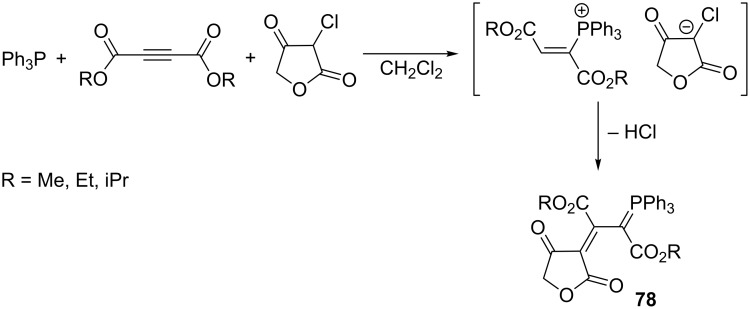
Synthesis of resonance-stabilized ylides in the reaction of triphenylphosphine, dialkyl acetylenedicarboxylates, and 3-chlorotetrahydrofuran-2,4-dione.

The syntheses of resonance-stabilized phosphorus ylides **78** derived from 3-chlorotetrahydrofuran-2,4-dione were carried out by Yavari and Nourmohammadian in good yields of 80–85% (Scheme 49) [22].

**Scheme 50:**
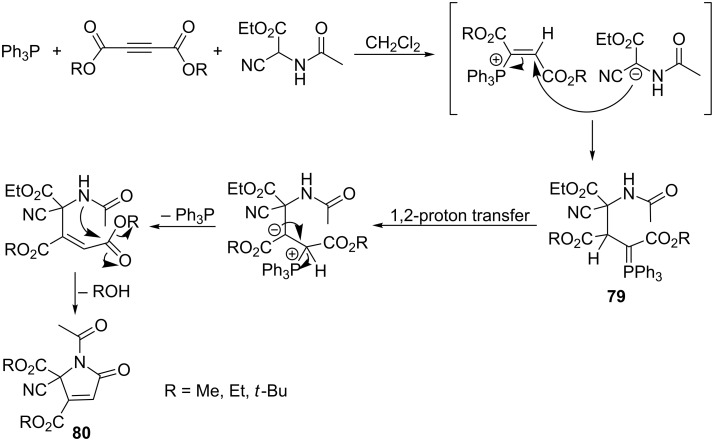
Synthesis of *N*-acylated α,β-unsaturated γ-lactams via resonance-stabilized phosphorus ylides derived from *N*-acetylaminocyanoacetate.

A facile route to *N*-acylated α,β-unsaturated γ-lactams **80** was reported by Asghari et al. The reaction of acetylenedicarboxylates with triphenylphosphine and *N*-acetylaminocyanoacetate gave the corresponding ylide **79**. The subsequent 1,2-proton transfer followed by the elimination of triphenylphosphine and ring closure via formation of the new C–N bond gave the final γ-lactam derivatives **80** (Scheme 50) [66].

**Scheme 51:**
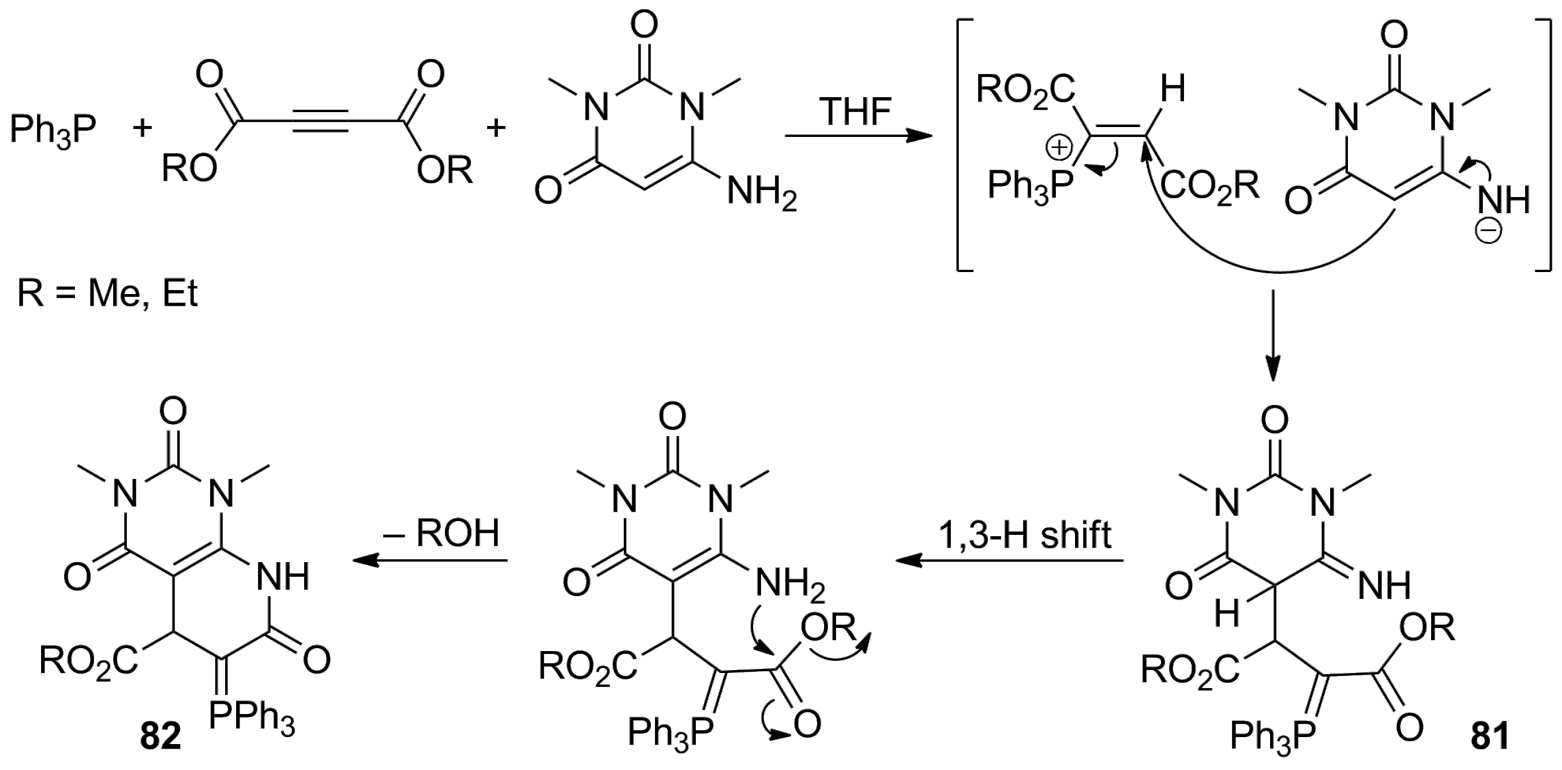
Synthesis of resonance-stabilized phosphorus ylides derived from 6-amino-*N*,*N'*-dimethyluracil and their subsequent cyclization to bicyclic compounds.

In 2013 Mohebat et al. used 6-amino-*N*,*N'*-dimethyluracil as an NH-acid and precursor of the carbon nucleophile in the synthesis of resonance-stabilized phosphorus ylides **81** (Scheme 51). The obtained ylides underwent further cyclization by the intramolecular acylation of the primary amino group to give the final bicyclic products **82** in 85–90% yields [67].

**Scheme 52:**
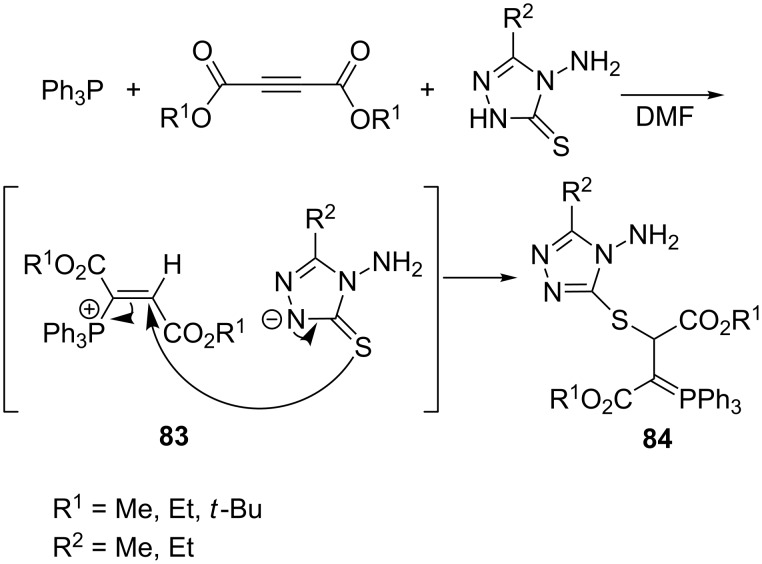
Generation of resonance-stabilized phosphorus ylides in the reaction of triphenylphosphine, dialkyl acetylenedicarboxylates, and 1,2,4-triazole-3-thione derivatives.

The synthesis of resonance-stabilized phosphorus ylides via the reaction of 4-amino-5-alkyl-2,4-dihydro-1,2,4-triazole-3-thione with DAAD and triphenylphosphine was reported by Mosslemin et al. [25]. The reaction of triphenylphosphine with DAAD and triazole as the NH-acid and a precursor of the ambident sulfur nucleophile gave the corresponding vinylphosphonium salt **83**. The further attack of the exocyclic sulfur atom of the triazolethione anion on the β-position of the vinylphosphonium salt led to the final phosphorus ylide **84** (Scheme 52). The above-mentioned three-component reaction was completed in a short period of time and allowed to obtain the final product in high yields of 92–97% [25].

**Scheme 53:**
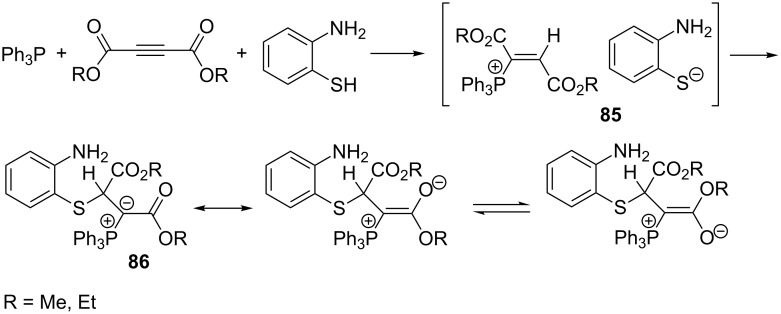
Synthesis of resonance-stabilized phosphorus ylides via the reaction of triphenylphosphine with dialkyl acetylenedicarboxylates and 2-aminothiophenol.

Esmaili et al. described the synthesis of functionalized, resonance-stabilized phosphorus ylides **86** from vinylphosphonium salt **85** generated in situ from triphenylphosphine, acetylenedicarboxylic acid ester, and 2-aminothiophenol in yields of 87–89% (Scheme 53) [23].

**Scheme 54:**
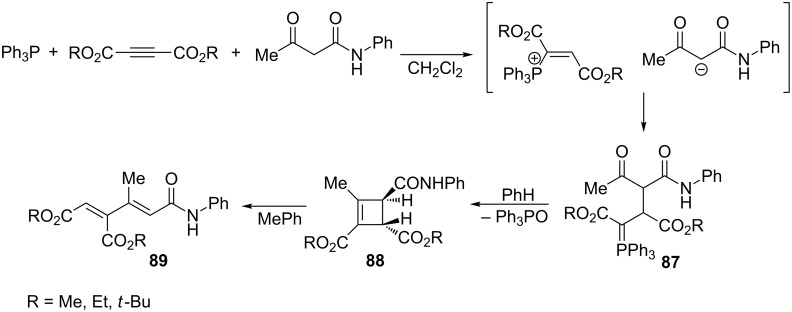
Synthesis of 1,3-dienes via intramolecular Wittig reaction with the use of resonance-stabilized ylides generated from acetylenedicarboxylates, triphenylphosphine, and acetoacetanilide.

There were several reported examples of intra- or intermolecular Wittig reactions of ylides generated from dialkyl acetylenedicarboxylate, triphenylphosphine, and some nucleophiles. Thus, Yavari and Asghari reported an interesting synthesis of highly electron-deficient 1,3-dienes in the reaction of acetoacetanilide with DAAD and triphenylphosphine. The obtained ylides **87** were first converted to cyclobutene derivatives **88** via the stereoselective intramolecular Wittig reaction. The resulting strained, four-membered cyclobutene derivatives underwent electrocyclic ring opening at the boiling point of toluene to the final 1,3-dienes **89** (Scheme 54) [21].

**Scheme 55:**
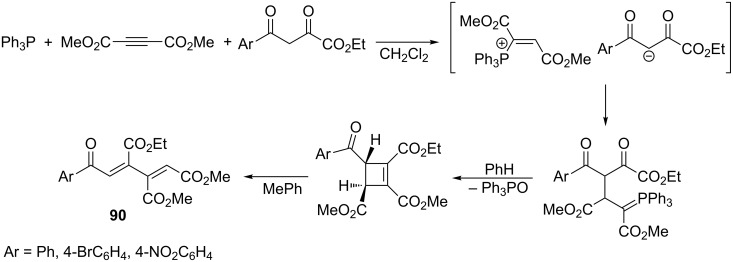
Synthesis of 1,3-dienes in the intramolecular Wittig reaction from ylides generated from dimethyl acetylenedicarboxylate, triphenylphosphine, and ethyl 4-aryl-2,4-dioxobutanoates.

A similar type of highly electron-deficient 1,3-dienes **90** were synthesized in yields of 78–87% in an analogous reaction with ethyl 4-aryl-2,4-dioxobutanoates with the corresponding vinylphosphonium salt (Scheme 55) [68].

**Scheme 56:**
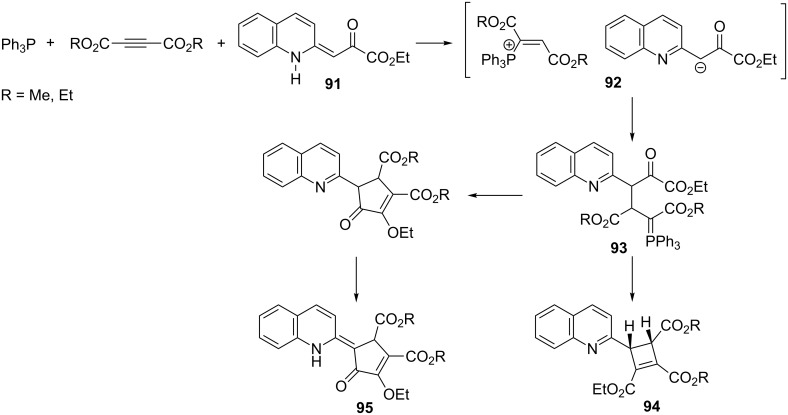
Synthesis of 4-(2-quinolyl)cyclobutene-1,2,3-tricarboxylic acid triesters and isomeric cyclopentenone derivatives via resonance-stabilized ylides in the intramolecular Wittig reaction.

In a similar reaction employing ethyl 3-(1,2-dihydroquinoline-2-ylidene)pyruvate (**91**), Yavari and co-workers obtained 4-(2-quinolyl)cyclobutene-1,2,3-tricarboxylic acid triesters **94** and isomeric cyclopentenone derivatives **95** with total yields of 87–94% and a ratio of 1:4 via the intramolecular Wittig reaction. The addition of triphenylphosphine to a triple bond of acetylenedicarboxylate followed by protonation of the adduct by the NH-acid gave the expected vinylphosphonium salt **92**. The attack of the carbanion on the β-position of the phosphonium salt followed by two competitive intramolecular Wittig reactions of the intermediate ylide **93** provided two final four- and five-membered reaction products **94** and **95**, respectively (Scheme 56) [69].

**Scheme 57:**
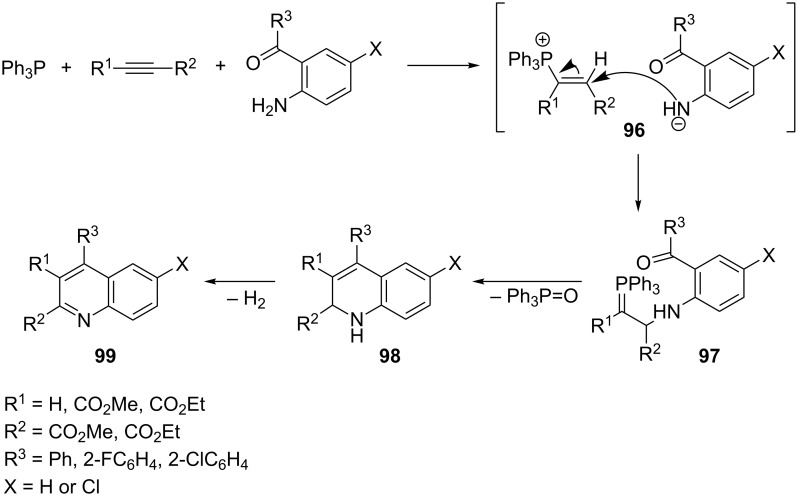
Synthesis of 4-arylquinolines via resonance-stabilized ylides in the intramolecular Wittig reaction.

Another example of the generation of heterocyclic compounds via the intramolecular Wittig reaction is the synthesis of quinoline derivatives **99** from triphenylphosphine, acetylenedicarboxylic acid esters, and aromatic 2-acylamines. The reaction of triphenylphosphine with DAAD and the 2-aminobenzophenone derivatives gave the vinylphosphonium salt **96** with a nucleophilic amide anion as a counterion. The attack of the nitrogen nucleophile on the β-position of the vinylphosphonium salt resulted in the expected ylide **97**. The subsequent intramolecular Wittig reaction in boiling toluene gave the 1,2-dihydroquinoline derivatives **98**, which underwent dehydrogenation into the corresponding 4-arylquinolines **99** in yields of 55–90% (Scheme 57) [70].

**Scheme 58:**
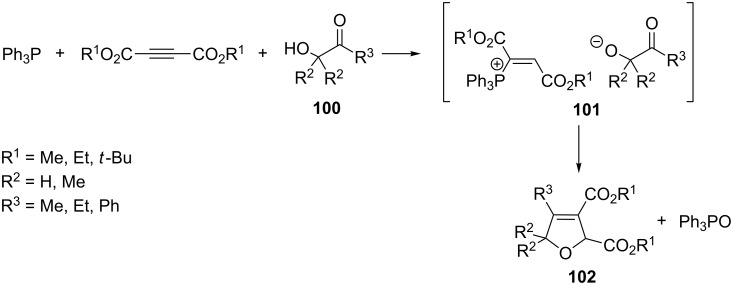
Synthesis of furan derivatives via resonance-stabilized ylides in the intramolecular Wittig reaction.

In an analogous manner, Yavari and Mosslemin synthesized furan derivatives from 2-hydroxyketones **100**, triphenylphosphine, and dialkyl acetylenedicarboxylates. The intermediate vinylphosphonium salt **101** was attacked here at the β-position by the nucleophilic oxygen atom of the hydroxyketone anion. The final product of this reaction was the expected furan derivative **102** that was formed by the intramolecular Wittig reaction at room temperature in CH_2_Cl_2_ in 70–81% yields (Scheme 58) [71].

**Scheme 59:**
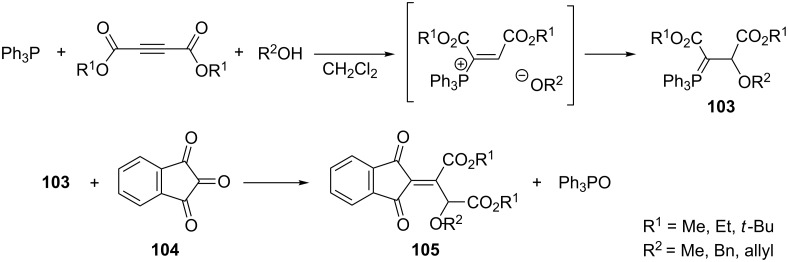
Synthesis of 1,3-indanedione derivatives via resonance-stabilized ylides in the intermolecular Wittig reaction.

An interesting example of employing in situ generated ylides in the intermolecular Wittig reaction was reported by Ramazani and Bodaghi. The addition of triphenylphosphine to a triple bond of acetylenedicarboxylic acid ester followed by the further attack of the alkoxyl anion on a vinylphosphonium cation provided the corresponding resonance-stabilized ylide **103**. The subsequent intermolecular Wittig reaction with the highly electron-deficient carbonyl group of indane-1,2,3-trione (**104**) led to the formation of 1,3-indanedione derivatives **105** in yields of 63–70% (Scheme 59) [72].

**Scheme 60:**
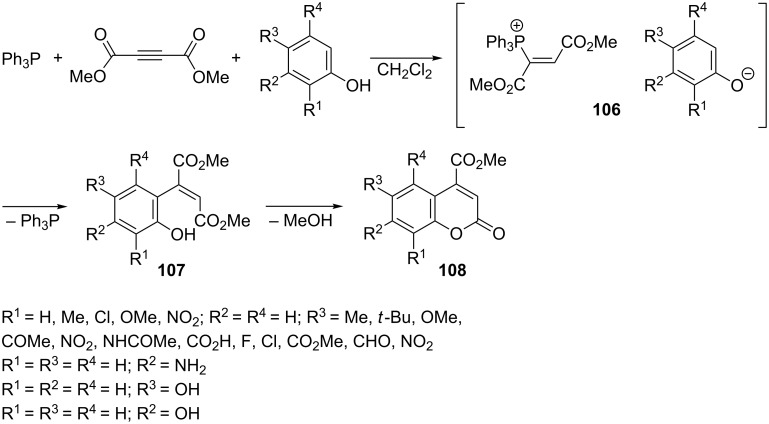
Synthesis of coumarin derivatives via nucleophilic displacement of the triphenylphosphonium group in intermediate α,β-di(alkoxycarbonyl)vinylphosphonium salts.

**Nucleophilic displacement of the triphenylphosphonium group in α,β-di(alkoxycarbonyl)vinylphosphonium salts:** Coumarins are an important class of natural compounds [73]. Yavari et al. developed a method for the synthesis of 4-methoxycarbonylcoumarins by reaction of dimethyl acetylenedicarboxylate and triphenylphosphine with substituted phenols. The vinyltriphenylphosphonium salt **106** that is formed as an intermediate product was attacked by the nucleophilic carbon atom of the phenolate anion, which led to the displacement of triphenylphosphine. Finally, coumarin derivatives **108** were obtained in yields of 40–90% as a result of intramolecular lactonization of the intermediate fumaric acid derivatives **107** [74]. In an analogous manner, but using 3-aminophenol, 7-aminocoumarin was obtained in a yield of 90% (Scheme 60) [63]. Similarly, 6-hydroxy- and 7-hydroxycoumarin derivatives were obtained from hydroquinone and resorcinol, respectively (Scheme 60) [75].

**Scheme 61:**
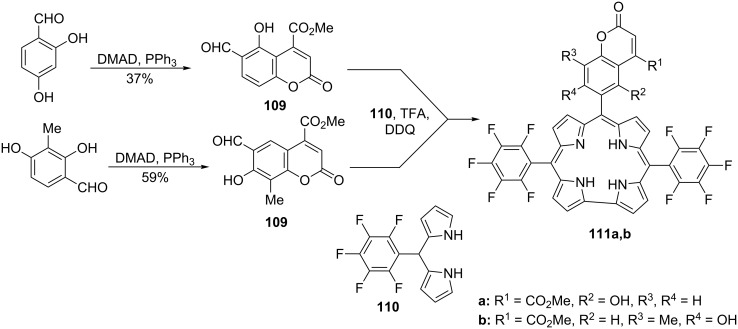
Synthesis of 6-formylcoumarin derivatives and their application in the synthesis of dyads.

The same kind of reaction with the participation of 2,4-dihydroxybenzaldehyde or 2,4-dihydroxy-3-methylbenzaldehyde was applied in 2010 by Gryko and Flamigni et al. for the preparation of 6-formylcoumarin derivatives **109** that are used in the synthesis of dyads **111** consisting of coumarin and corrole units (Scheme 61). The latter synthesis took place by condensation of formylcoumarins **109** with 5-(pentafluorophenyl)dipyrromethane (**110**) [76].

**Scheme 62:**
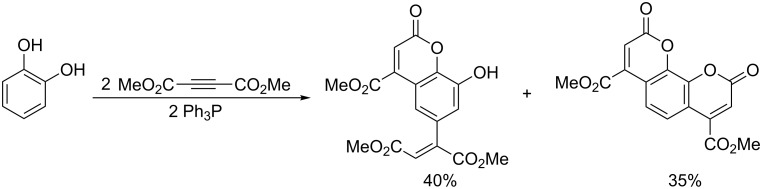
Synthesis of di- and tricyclic coumarin derivatives in the reaction of pyrocatechol with two vinylphosphonium salt molecules.

**Scheme 63:**
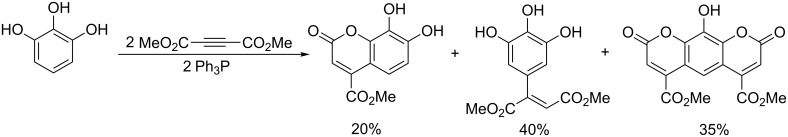
Synthesis of mono-, di-, and tricyclic derivatives in the reaction of pyrogallol with one or two vinylphosphonium salt molecules.

The use of pyrocatechol or pyrogallol as reagents in reactions with the analogous mechanism resulted in the formation of mono-, di-, and tricyclic reaction products of phenol derivatives with one or two vinylphosphonium salt molecules (Scheme 62 and Scheme 63) [75].

**Scheme 64:**
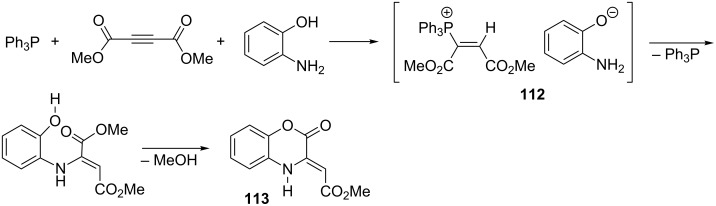
Synthesis of 1,4-benzoxazine derivative by nucleophilic displacement of the triphenylphosphonium group in intermediate α,β-di(methoxycarbonyl)vinylphosphonium salt.

In a similar reaction with 2-aminophenol, 1,4-benzoxazine derivative **113** was obtained in a yield of 70% [63]. According to the authors, the displacement of the triphenylphosphonium group resulted from the nucleophilic attack of the amine group of 2-aminophenol on the β-position of the vinylphosphonium salt **112**, followed by the 1,2-cationotropic proton shift to the α-position and elimination of triphenylphosphine (Scheme 64) [63]. It seems, however, that the direct displacement of the triphenylphosphonium group by the attack of the amine group on the α-position of the vinylphosphonium salt is more probable.

**Scheme 65:**
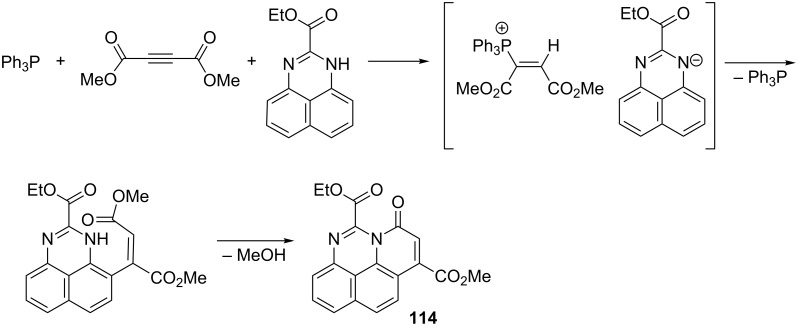
Synthesis of 7-oxo-7*H*-pyrido[1,2,3-*cd*]perimidine derivative via nucleophilic displacement of the triphenylphosphonium group in intermediate α,β-di(methoxycarbonyl)vinylphosphonium salt.

In an analogous manner, the reaction of ethyl 1*H*-perimidine-2-carboxylate with acetylenedicarboxylic acid ester and triphenylphosphine provided the 7-oxo-7*H*-pyrido[1,2,3-*cd*]perimidine derivative **114** in a yield of 70% (Scheme 65) [77].

**Scheme 66:**
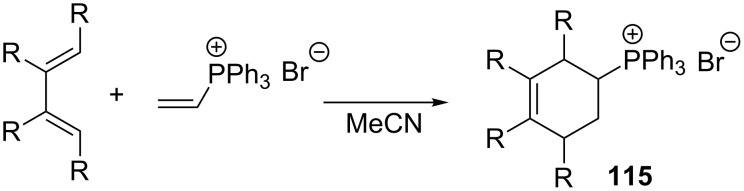
Application of vinylphosphonium salts in the Diels–Alder reaction with dienes.

**2.3.3. Other reactions of vinylphosphonium salts:** Bonjouklian and Ruden demonstrated that vinylphosphonium salts can be used in the Diels–Alder reaction with dienes such as isoprene, 1,3-butadiene, 2,3-dimethyl-1,3-butadiene, cyclopentadiene, and 1,3-cyclohexadiene, resulting in cyclic phosphonium salts **115** in yields of 90–96% (Scheme 66) [78].

**Scheme 67:**
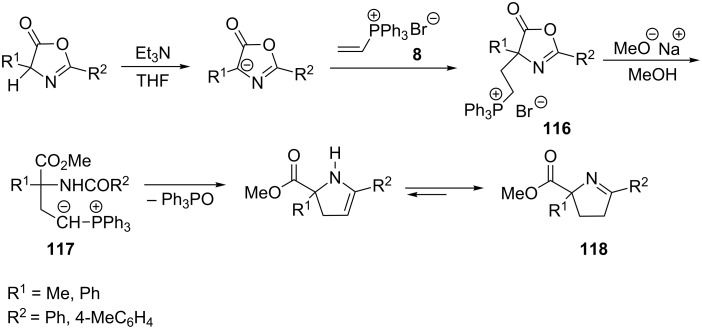
Synthesis of pyrroline derivatives from vinylphosphonium bromide and 5-(4*H*)-oxazolones.

Gelmi et al. showed that vinylphosphonium bromide **8** in reaction with 5-(4*H*)-oxazolones in the presence of triethylamine formed 4-(2-phosphonioethyl)-5(4*H*)-oxazolones **116**. The oxazolone ring opening with methanol and subsequent deprotonation with sodium methoxide converted phosphonium salt **116** into phosphorous ylide **117**, which underwent an intramolecular Wittig reaction that eventually led to pyrroline derivatives **118** (Scheme 67) [79].

**Scheme 68:**
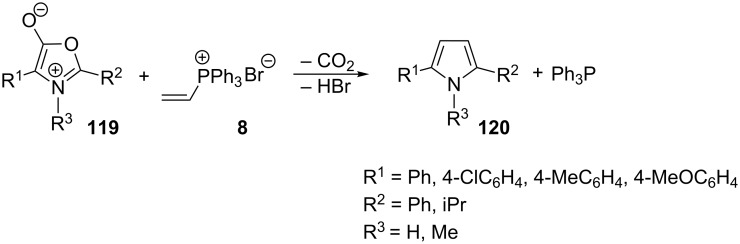
Synthesis of pyrrole derivatives in the reactions of vinyltriphenylphosphonium bromide with protonated 5-(4*H*)-oxazolones or münchnones.

Gelmi et al. also conducted a number of syntheses of pyrrole derivatives **120** from vinylphosphonium bromide **8** and protonated 5-(4*H*)-oxazolones **119** (Scheme 68, R^3^ = H) as well as the so-called münchnones **119** (Scheme 68, R^3^ = Me). The reaction yields for the protonated oxazolones were ca. 40% and 48–53% in the reactions involving münchnones (Scheme 68) [80].

**Scheme 69:**
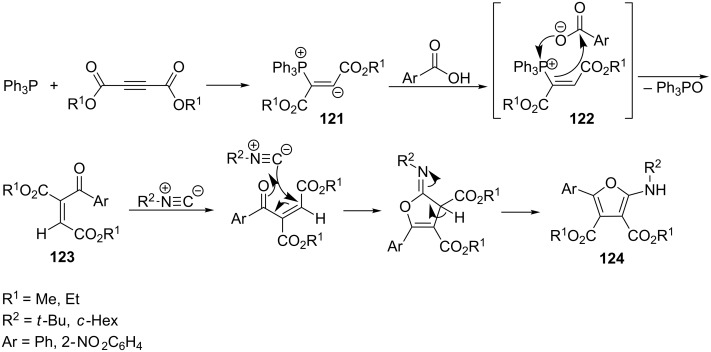
Synthesis of dialkyl 2-(alkylamino)-5-aryl-3,4-furanedicarboxylates via intermediate α,β-di(alkoxycarbonyl)vinylphosphonium salts.

In 2007 Alizadeh et al. described an interesting reaction of α,β-di(alkoxycarbonyl)vinylphosphonium salts with the carboxylate anion. The reaction of triphenylphosphine with dialkyl acetylenedicarboxylate followed by protonation of the adduct **121** by carboxylic acid provided the α,β-di(alkoxycarbonyl)vinylphosphonium salt **122** with the carboxylate anion as a counterion. The nucleophilic carboxylate anion then attacked the phosphorus atom of the vinylphosphonium salt cation, resulting in the removal of triphenylphosphine oxide and the formation of a new C–C bond between the carbon atom at the α-position of the phosphonium salt and the carbon atom of the carbonyl group of the carboxylate anion. The obtained enone **123** was then trapped by reaction with alkyl isocyanide, which finally led to ring closure and the formation of dialkyl 2-(alkylamino)-5-aryl-3,4-furanedicarboxylate **124** in a yield of 69–88% (Scheme 69). The reaction was carried out for several hours at room temperature in anhydrous CH_2_Cl_2_ [81].

**Scheme 70:**
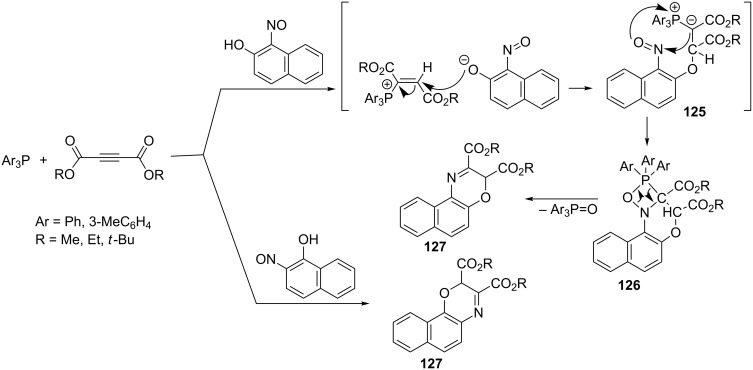
Synthesis of 1,4-benzoxazine derivatives from acetylenedicarboxylates, phosphines, and 1-nitroso-2-naphthol or 2-nitroso-1-naphthol in the intramolecular Wittig-like cyclization.

Recently, an interesting one-pot condensation of acetylenedicarboxylates with phosphines and 1-nitroso-2-naphthol or 2-nitroso-1-naphthol leading to 1,4-benzoxazine derivatives was reported. The addition of triarylphosphine to an acetylenic ester followed by protonation of the adduct by the naphthol derivative and the further attack of the resulting naphtholate anion on the β-position of the vinylphosphonium cation gave the corresponding ylide **125**. The intramolecular Wittig-like cyclization of the ylide **125** via the intermediate oxazaphosphetane **126** provided the final 1,4-benzoxazine derivatives **127** in good yields of 72–87% (Scheme 70) [82].

## Conclusion

Easily accessible vinylphosphonium salts are important reagents and building blocks in organic synthesis mainly due to the convenience of their transformation into reactive ylides by addition of a variety of nucleophiles, including carbon, nitrogen, sulfur, and oxygen nucleophiles. The addition of a bifunctional nucleophile with a carbonyl function to vinylphosphonium salts can be considered as a general method for a new ring closure to carbo- or heterocyclic systems of diversified size by the intramolecular Wittig reaction. Recently, significant attention attracts also highly reactive α,β-(dialkoxycarbonyl)vinylphosphonium salts, generated easily in situ from acetylenedicarboxylic acid diester, triarylphosphine and a nucleophile. Depending on the structure of the nucleophile used, the salts convert into the corresponding resonance-stabilized, relatively stable ylides or undergo intramolecular nucleophilic substitution with triphenylphosphine departure, usually followed by cyclization with the engagement of one of the alkoxycarbonyl groups to carbo- or hetereocyclic systems. 2-Aminovinylphosphonium salts have also found several interesting synthetic applications, although they have become known relatively recently.
